# Review of evolution of the public long-term care insurance (LTCI) system in different countries: influence and challenge

**DOI:** 10.1186/s12913-020-05878-z

**Published:** 2020-11-20

**Authors:** Linhong Chen, Lu Zhang, Xiaocang Xu

**Affiliations:** 1 School of Mathematics and Statistics, Chongqing Technology and Business University, Chongqing, 400067 China; 2 School of Public Administration, Sichuan University, Chengdu, 610065 China; 3 Research Center for Economy of Upper Reaches of the Yangtse River, Chongqing Technology and Business University, Chongqing, 400067 China; 4 School of Economics, Chongqing Technology and Business University, Chongqing, 400067 China; 5 Department of Actuarial Studies & Business Analytics, Macquarie University, Sydney, 2109 Australia

**Keywords:** LTC (long-term care), Public LTCI (long-term care insurance), Economic impact, Financial burden, Challenge

## Abstract

**Background:**

The growing demand for LTC (Long-term care) services for disabled elderly has become a daunting task for countries worldwide, especially China, where population aging is particularly severe. According to CSY (China Statistical Yearbook,2019), the elderly aged 65 or above has reached 167 million in 2018, and the number of disabled elderly is as high as 54%. Germany and other countries have alleviated the crisis by promoting the public LTCI (Long-Term Care Insurance) system since the 1990s, while China’s public LTCI system formal pilot only started in 2016. Therefore, the development of the public LTCI system has gradually become a hot topic for scholars in various countries, including China.

**Methods:**

This review has been systematically sorted the existing related literature to discuss the development of public LTCI (Long-Term Care Insurance)system form four aspects, namely, the comparison of public LTCI systems in different countries, the influence of public LTCI, challenge of public LTCI, and the relationship between public LTCI and private LTCI. We searched some databases including Web of Science Core Collection, Medline, SCOPUS, EBSCO, EMBASE, ProQuest and PubMed from January 2008 to September 2020. The quality of 38 quantitative and 21 qualitative articles was evaluated using the CASP(Critical Appraisal Skills Programme) critical evaluation checklist.

**Results:**

The review systematically examines the development of public LTCI system from four aspects, namely, the comparison of public LTCI systems in different countries, the influence of public LTCI, the challenge of public LTCI, and the relationship between public LTCI and private LTCI. For example, LTCI has a positive effect on the health and life quality of the disabled elderly. However, the role of LTCI in alleviating the financial burden on families with the disabled elderly may be limited.

**Conclusion:**

Some policy implications on the future development of China’s LTCI system can be obtained. For example, the government should fully consider the constraints such as price rise, the elderly disability rate, and the substantial economic burden. It also can strengthen the effective combination of public LTCI and private LTCI. It does not only help to expand the space for its theoretical research but also to learn the experiences in the practice of the LTCI system in various countries around the world. It will significantly help the smooth development and further promote the in-depth reform of the LTCI system in China.

## Background

The aging of the population has become a global problem. According to the World Population Outlook (2019) (WPP2019, United Nations Population Division), the Aging degree (proportion of the population aged 65 and above) of Japan, Italy, South Korea, and China will reach 28.4, 23.3, 15.8, and 12.0% respectively in 2020, while by 2050, the Aging degree of South Korea, Japan, Italy, and Germany will reach 38.1, 37.7, 36.0, and 30.0% respectively, and China will surge to 26.1%, which is equivalent to the average level of developed countries (26.9%). Subsequently, the cost of long-term care for the elderly who have lost their Activities of Daily Living (ADL) will bring a heavy economic burden to their families [1]. Therefore, the Long-term care insurance (LTCI) system came into being [2].

Long term care insurance (LTCI) system refers to an institutional arrangement to share the nursing expenses incurred by people who cannot take care of themselves due to chronic diseases or physical and psychological disability (WHO) [3]. From the perspective of whether it is compulsory or not, Long term care insurance (LTCI) system includes public-LTCI and private-LTCI. Private-LTCI exists as a supplement to public-LTCI, and its development is seriously insufficient. Therefore, at present, the focus of government and academic discussion is in the field of public-LTCI, which is also the main research object of this paper.

### Practice of long-term care insurance (LTCI) system in different countries

Long-term care insurance (LTCI) system originated in European countries. The Netherlands was the first country that introduced a universal mandatory social health insurance scheme for covering a broad range of long-term care (LTC) services provided in a variety of care settings in the 1960s. Germany is the first country to implement public LTCI(Long-term care insurance) in the form of social legislation. In 1995, LTCI law came into effect and became the fifth pillar insurance after endowment insurance, medical insurance, accidental injury insurance, and unemployment insurance. Since then, the United States and other countries have also formally enacted public LTCI.

Japan and South Korea are the first countries to implement public LTCI in Asia. After the implementation of public LTCI system in Japan since 2000, it has gone through five major changes (2000, 2004, 2006, 2009, 2014), and has a careful design in the funding sources, identification procedures, and service content. According to the Japanese “ LTCI law”, the insured only pays 10% of the total nursing expenses when receiving long-term care services, and the remaining expenses are borne by 50% of the premium paid by the insured and 50% of the government’s public expenses. This way of financing can ensure that the family’s economic burden is not too heavy and not cause too much financial pressure, and ensure that the public LTCI system can be carried out persistently and stably. Korea has also implemented public LTCI to maintain and improve the health and well-being of the elderly since 2008.

The increasingly aging population has highlighted the urgency of the crisis in health care services for the elderly in China in recent years. According to CSY (China Statistical Yearbook,2019), the elderly aged 65 or above has reached 167 million in 2018, accounting for 11.9% of the total population. The ODR (Old-age dependency ratio) has climbed from 9.9% in 2000 to 16.8% in 2018. Among them, the number of disabled elderly due to chronic diseases, environmental pollution, accidental injuries, and natural aging is increasing, and the prevalence rate of chronic diseases in the elderly is as high as 54% [4–8]. Although the LTC (Long-Term Care) services demand for the elderly was snowballing, the provision of LTC (Long-Term Care) services such as policy formulation, operational models, and especially fundraising lag far behind. As a result, the “healthy China strategy” has been proposed in the Report of the 19th National Congress of China. At the same time, Government-mandated public LTCI (Long-Term Care Insurance) was piloted in 15 cities since 2016, including Qingdao of Shandong province, Chengdu of Sichuan province, and Chongqing, which had a profound impact on the people and economies of the pilot areas(as shown in Table 1).

**Table 1:** The first 15 public LTCI pilot cities in China since 2016

***City***	***Start Time***	***City***	***Start Time***
Changshu	October 2018	Changchun	May 2015
Chengdu	July 2017	Jingmen	December 2016
Ningbo	December 2017	Nantong	January 2017
Chongqing	March 2018	Qinghai	July 2012
Anqing	January 2017	Shanghai	January 2018
Chengde	July 2017	Shangrao	November 2016
Qiqihaer	October 2017	Shihezi	January 2017
Guangzhou	April 2017		

### Academic study of long-term care insurance (LTCI) system in different countries

Academic research on Long-term care insurance is carried out from two main lines: public LTCI and private LTCI. The research on private LTCI mainly focuses on the design of different types of LTCI insurance and people’s willingness to purchase private LTCI [9]. The research on public LTCI mainly focuses on two aspects. One is how to optimize the financing method to ensure the public LTCI can be carried out continuously and stably. The second is to discuss the effect of LTCI implementation on the health or financial burden of people who benefit from LTCI. For example, Choi & Joung used Cox proportional risk regression model to prove that LTCI (Long-Term Care Insurance) services can help reduce health expenditure and protect the health of the elderly aged 65 and above [10]. All the studies show that LTCI has a positive impact on the health of the disabled elderly, and the theoretical research is becoming more and more in-depth, which were conducted from the perspectives of different age groups, nursing time, and health recovery degrees. Etc.

Looking at the historical evolution of the Long-term care insurance system, we can find that although private LTCI is an important aspect of LTCI system, it is essentially only a supplement to public LTCI. Therefore, this paper only takes public LTCI as the research object to discuss the development of public LTCI system form four aspects, namely, the comparison of public LTCI systems in different countries, the influence of public LTCI, challenge of public LTCI, and the relationship between public LTCI and private LTCI.

## Methods

The review aimed to show the influence and challenge of the public Long-Term Care Insurance (LTCI) System in Different Countries. By the way, we also discussed the development process of public LTCI in major countries (such as Germany, Japan, South Korea, and China) and its relationship with private LTCI.

### Search process

The following main databases were taken into account: Web of Science Core Collection, Medline, SCOPUS, EBSCO, EMBASE, ProQuest and PubMed. Considering the regional limitations and the disorder of Chinese literature, CNKI(China) was not included in this literature search. The following combinations of terms were practiced with the Boolean phrase “and/or” to maximize the scope and type of material achieved in the search: ‘LTCI’ OR “Long Term Care Insurance”.

### Inclusion and exclusion criteria

By September 30, 2020, we have searched for a total of 445 papers. The selection criteria for publications were as follows: (1) In terms of publication time, we limit it from 2008 to September 30, 2020 (the time of revision of this paper). Although the ‘Long Term Care Insurance’ article first appeared back in 1984 (Meiners&Tave.1984 [11]; Schechter,1984 [12]; Meiners&Trapnell,1984 [13]), but they simply put forward the concept of ‘Long Term Care Insurance’, and there were few studies on the subject over the next 25 years. It was not until 2008 that Brown & Finkelstein (2008) [14] first discussed the relationship between American public LTCI and private LTCI, and Ariizumi(2008) [15] first discussed the effect of public long-term care insurance on consumption and welfare, that the topic of long term care insurance began to attract the attention of a large number of scholars. Therefore, this paper takes 2008 as the starting point of research time. (2) The following types of documents are not included: Meeting abstract, Editorial Material, Letter, Proceedings paper, and other types such as Opinions or Comments (194 articles in total). In the end, we determined a total of 59 articles, including 38 quantitative research and 21 qualitative research.

### Data extraction process

All publication information was exported to the Excel database via Endnote, and duplicate sections were removed. The results were initially extracted by one researcher and then cross-checked by another to ensure that all data had been screened and reviewed. If there is a difference of opinion between the two researchers, the third researcher will be invited to express his opinion and finally reach an agreement.

**Fig. 1:**
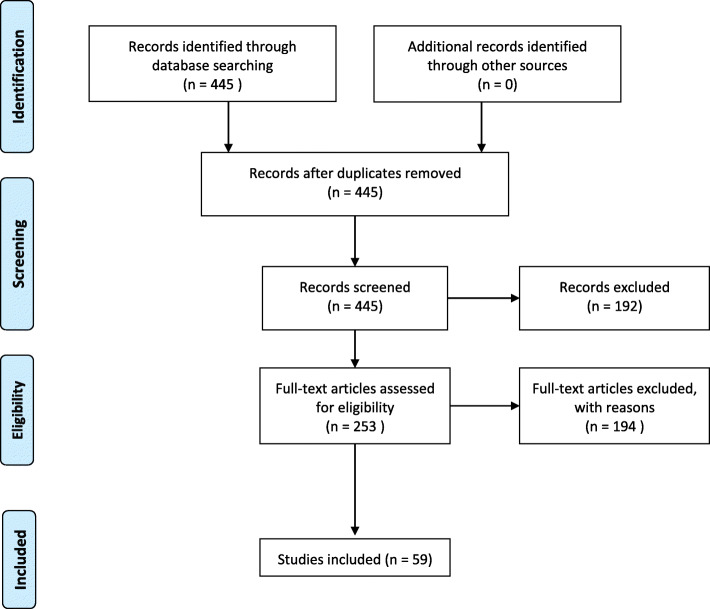
Data extraction process (PRISMA)

The information extracted from all the included publication was as follows: Author, Publication date, Sample country, Research method, Research objective, and Key findings. All the analysis results are analyzed in the following process (as shown in Fig. 1).

### Quality appraisal

We used the Critical Appraisal Skills Programme (CASP) to evaluate all 59 papers that were selected and selected 4 of the original 10 questions within the CASP Checklist that was suitable for our paper to evaluate the quality of the paper (shown in Table 2). The five questions are: (1)Did the paper address a clearly focused question? (2)Do you think all the important, relevant studies were included? (3) Can the results be applied to the local population? (4) Were all important outcomes considered? All the questions were asked to answer one of the following answers: Yes; No; Can’t Tell. The evaluation results of all literatures are shown in Table 2.

**Table 2:** Quality Assessment of all studies

***Rank***	***Author***	(1)Did the paper address a clearly focused question?	(2)Do you think all the important, relevant studies were included?	(3) Can the results be applied to the local population?	(4) Were all important outcomes considered?
1	Brown&Finkelstein (2008) [14]	●	**※**	●	**△**
2	Ariizumi (2008) [15]	●	**※**	●	●
3	Yamada et al. (2009) [16]	**△**	**●**	●	●
4	Ohwaki et al. (2009) [17]	●	**※**	●	**※**
5	Yoshioka et al. (2010) [18]	**※**	**●**	●	**※**
6	Campbell, et al. (2010) [19]	●	**●**	●	●
7	Seok(2010) [20]	●	**※**	●	●
8	Iwamoto et al. (2010) [21]	●	**※**	●	**△**
9	Buscher et al. (2010) [22]	●	**●**	●	●
10	Schut& van den Berg (2010) [23]	●	**●**	●	**※**
11	Rothgang (2010) [24]	●	**△**	●	●
12	Zuchandke et al. (2010) [25]	●	**●**	●	●
13	Costa-Font (2010) [26]	**△**	**※**	●	**△**
14	Tomita et al. (2010) [27]	●	**※**	●	**※**
15	Tamiya et al. (2011) [28]	●	**※**	●	●
16	Kang et al. (2012) [29]	●	**△**	●	●
17	Schwarzkopf et al. (2012) [30]	●	**※**	●	**※**
18	Olivares-Tirado et al. (2012) [31]	●	**※**	●	**△**
19	Washio et al. (2012) [32]	●	**●**	●	●
20	Chon (2012) [33]	**※**	**△**	●	**△**
21	Chen et al. (2013) [34]	●	**●**	●	**※**
22	Chon (2013) [35]	●	**※**	●	●
23	Kim& Choi (2013) [36]	●	**※**	●	**△**
24	Kim et al. (2013) [37]	●	**●**	●	●
25	Umegaki et al. (2014) [38]	**△**	**※**	●	**△**
26	Shen et al. (2014) [39]	●	**※**	●	**※**
27	Lee et al. (2014) [40]	●	**※**	●	●
28	Cremer&Pestieau(2014) [41]	●	**△**	●	●
29	Chon(2014) [42]	●	**※**	●	**※**
30	Hyun et al. (2014) [43]	●	**※**	●	**△**
31	Costa-Font&Courbage (2015) [44]	●	**※**	●	●
32	Kim& Lim (2015) [45]	**△**	**●**	●	●
33	Bakx et al. (2015) [46]	●	**※**	●	**※**
34	Bakx,&Schut&van Doorslaer (2015) [47]	**※**	**●**	●	**※**
35	Geyer&Korfhage (2015) [48]	●	**●**	●	●
36	Park (2015) [49]	●	**※**	●	●
37	Rhee et al. (2015) [2]	●	**※**	●	**△**
38	Strier&Werner (2016) [50]	●	**●**	●	●
39	Ha et al. (2017) [51]	●	**※**	●	●
40	Bascans et al. (2017) [52]	●	**※**	●	●
41	Geyer et al. (2017) [53]	●	**※**	●	**※**
42	Fu et al. (2017) [54]	●	**●**	●	**△**
43	Wang et al. (2018) [55]	●	**●**	●	**※**
44	Saito et al. (2018) [56]	●	**※**	●	●
45	Nadash et al. (2018) [57]	**※**	**△**	●	**※**
46	Ayalon (2018) [58]	●	**※**	●	●
47	Kato (2018) [59]	●	**※**	●	●
48	Choi et al. (2018) [60]	●	**※**	●	●
49	Schmitz&Giese (2019) [61]	●	**●**	●	●
50	Kim et al. (2019) [62]	●	**●**	●	●
51	Zhu&Osterle (2019) [63]	**※**	**△**	●	**△**
52	Kondo (2019) [64]	●	**●**	●	**※**
53	Zhang&Yu (2019) [65]	●	**※**	●	●
54	Sohn et al. (2020) [66]	●	**※**	●	**△**
55	Yang et al. (2020) [67]	●	**※**	●	**※**
56	Zhang et al. (2020) [68]	**※**	**●**	●	**※**
57	Courbage et al. (2020) [69]	●	**●**	●	●
58	Chandoevwit&Wasi (2020) [70]	●	**※**	●	●
59	Feng et al. (2020) [71]	●	**※**	●	**△**

● Yes; **△** No; ※Can’t Tell

## Results

### Preliminary review of relevant literature

Of the 59 papers selected, 38 were quantitative and 21 were qualitative. Among them, quantitative research is mainly carried out from two perspectives. One is empirical research through microscopic investigation data, such as logistic regression analysis. The second is to discuss the long-term sustainable development of public LTCI caused by financing problems by comparing the financing of public LTCI with the macro data of cost demand.

**Fig. 2:**
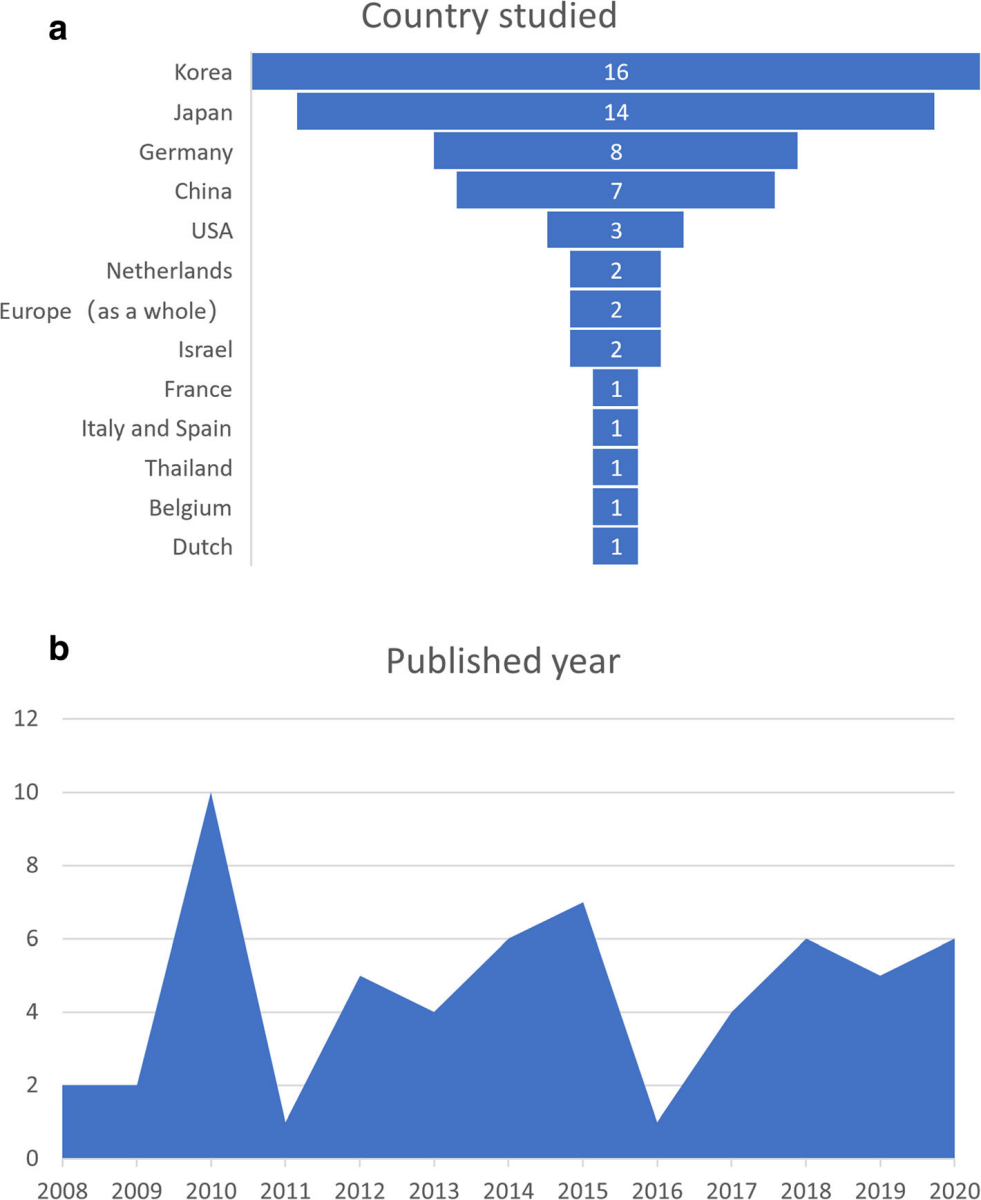
Classification of publication by country studied and publication year

The publication time and sample countries of public LTCI thematic research are closely related to the development experience of public LTCI in each country. Figure 2 shows some information.

As shown in Fig. 2(a), the research on public LTCI is mainly concentrated in Korea(16), Japan (14), and Germany(8). Germany is the first country in the world to formally legislate public LTCI (1995), while Japan (2000) and South Korea (2008) are the first countries in Asia to formally legislate public LTCI. In recent years, these three countries have been carrying out many reforms to different degrees for public LTCI according to their national conditions. It is worth noting that since China began to pilot public LTCI in 15 cities in 2016, relevant research has begun to emerge. In addition, the research on Thailand’s public LTCI began to appear. Although Thailand’s public LTCI system has not been formally implemented, it at least shows that Thailand is trying to make relevant efforts. The information is given in Fig. 2(b) is also very interesting. It shows that the research on public LTCI can be divided into three climaxes in terms of time, namely, 2009–2010, 2014–2015, and 2017–2019.

### Comparison of public LTCI systems in different countries

As early as 10 years ago, Campbell et al. (2010) [2] compared the differences between Germany and Japan in the objectives, qualification procedures, scale, and sustainability of the public LTCI system, hoping to learn from the experience and find a suitable LTCI system for the American situation. Bakx et al. (2015) [2] used data from the Netherlands and Germany, two countries with general public LTCI, and found that although the overall use rate of LTC was similar in the two countries, the use of formal care was more common in the Netherlands and the use of informal care was more common in Germany. Courbage et al. (2020) [2] also performed similar comparative studies on Italy and Spain through cross-sectional data from the European Health, Ageing, and Retirement Survey (SHARE) database. Rhee et al. (2015) [2] compared the public LTCI systems of Korea, Japan, and Germany from the perspective of financing, and believed that in order to achieve the sustainable development of LTCI, the financing of LTCI should be planned in advance in terms of income generation, welfare design, and qualification. A more meaningful study was conducted by Chandoevwit&Wasi (2020) [2], who discussed the feasibility and benefits of introducing public LTCI to middle-income countries such as Thailand through discrete Choice experiment (DCEs). To sum up, the comparison of public LTCI system in different countries is mainly carried out from the three aspects of structure design, fund raising and policy effect of public LTCI system (as shown in Table 3).

**Table 3:** The Comparison of public LTCI systems in different countries

Rank	Author	Journal	Country	Method	Objective / feature	Key findings
1	Campbell, et al. (2010) [19]	HEALTH AFFAIRS	Germany, Japan, and the United States	Qualitative research	It explored differences between Germany and Japan in program goals, eligibility process, scope, size, and sustainability for possible applications in the United States.	Public spending on long-term care in the United States is actually higher than in Germany, and is only slightly lower than in Japan.
2	Bakx&De Meijer& Van Doorslaer (2015) [46]	HEALTH ECONOMICS	Netherlands and Germany	Quantitative research	Using comparable data from two countries with universal public LTC insurance, the Netherlands and Germany, we examine how institutional differences relate to differences in the choice for informal and formal LTC.	System features such as eligibility rules and coverage generosity and, indirectly, social preferences can influence the choice between formal and informal care. Less comprehensive coverage also has equity implications: for the poor, access to formal LTC is more difficult in Germany than in the Netherlands.
3	Rhee et al. (2015) [2]	HEALTH POLICY	Korea, Japan and Germany	Qualitative research	This paper examined the financing systems of long-term care insurance (LTCI) in South Korea, Japan, and Germany.and draw lessons regarding revenue generation, benefits design, and eligibility.	1. Early financing ensures that the service delivery system has time to adapt because most middle-income countries lack the infrastructure for providing long-term care services. 2.One approach is to start with a limited benefit package and strict eligibility rules and expanded the program as the country develops sufficient experience and more providers became available.
4	Courbage et al. (2020) [69]	EUROPEAN JOURNAL OF HEALTH ECONOMICS	Italy and Spain	Quantitative research	This article test the effect of both long-term care (LTC) public benefits and insurance on the receipt of informal care provided by family members living outside the household in Italy and Spain.	LTC public support decreasing the receipt of informal care for Spain while reject it for Italy. They tend to confirm that the effect of public benefits on informal care depends on the typology of public coverage for LTC whereby access to proportional benefits negatively influences informal care receipt while access to cash benefits exerts a positive effect.
5	Chandoevwit,&Wasi,(2020) [70]	SOCIAL SCIENCE & MEDICINE	Thailand	Quantitative research	using Discrete choice experiments (DCEs) to evaluate the benefits of introducing a public long-term care insurance program to a middle-income country, Thailand.	when designing a new program, translating preference information into the demand for packages and benefits of alternative schemes (the choices made available) can make the DCE results more policy relevant.

### Influence of public LTCI

The social and economic impact of public LTCI on population aging is enormous, and many scholars have analyzed its influence from different angles (as shown in Table 4).

**Table 4:** The Influence of public LTCI

Rank	Author	Journal	Country	Method	Objective / feature	Key findings
***1. the impact of public LTCI on the health of beneficiaries***						
1	Buscher,et al. (2010) [22]	ZEITSCHRIFT FUR GERONTOLOGIE UND GERIATRIE	Germany	Qualitative research	qualitative interviews were conducted with family caregivers and on the general performance of the visits, the perception of care recipients and their family members, and from the perspective of the nursing services.	Based on the findings, a family-oriented approach for home care counseling was developed and evaluated in 80 home care arrangements. The physical health of the beneficiaries of the public LTCI has improved significantly for the most part, especially those receiving good home care.
2	Olivares-Tirado et al. (2012) [31]	BMC HEALTH SERVICES RESEARCH	Japan	Quantitative research	This paper discussed the effect of LTCI services on the progression of recipient disability in Japan since 2000.	1.women had a significantly greater probability of improving their functional status during all phases of the observation period. 2.disability transition as a measure of disability progression may not be specific enough to assess changes in functional status of LTCI recipients.
3	Ohwaki, et al. (2009) [17]	AGING CLINICAL AND EXPERIMENTAL RESEARCH	Japan	Quantitative research	This study was to examine the impact of social engagement and other predictive factors, including disability, household composition, and formal services, on continuity in home care of the elderly.	Participants who lived alone or with a spouse were less likely to continue to receive home care compared with those who lived with others. Having friends was a significant predictor of continuity in home care. The promotion of social engagement may be important in preventing institutionalization.
4	Chen et al. (2013) [34]	GERIATRICS & GERONTOLOGY INTERNATIONAL	Japan	Quantitative research	This study show which dimensions of functions differ among community-dwelling elderly participants in four different certification levels of the current long-term care insurance system (LTCI) in Japan.	Exercise and drinking habits were significantly less common in support- or care-level elderly than in specified or uncertified elderly. The prevalence of taking antihypertensive, antihyperlipidemic, antidepressant or sleeping medications was significantly higher in the support- or care-level elderly than in uncertified or specified elderly people. Support- or care-level elderly also had a significantly higher prevalence of past medical histories of stroke, bone fractures, osteoarthropathy, heart disease and cancer than uncertified or specified elderly people.
5	Lee et al. (2014) [40]	JOURNAL OF THE AMERICAN GERIATRICS SOCIETY	Korea	Quantitative research	This paper examined the effect of LTCI service type on the cognitive function, behavioral symptoms, and physical function.	There were significant differences in cognitive function, behavioral symptoms, and physical function at baseline between individuals receiving the three LTCI service type (HC, IC, CC) and overall improvements in those outcomes over 2 years in the three groups.
6	Sohn et al. (2020) [66]	SOCIAL SCIENCE & MEDICINE	Korea	Quantitative research	This study aimed to evaluate the effects of LTCI on mortality of elders in South Korea.	1.the disparities in mortality by income gap in in-home care users of LTCS was greater than that of facility care users in Korea. 2.Ensuring a “continuum of care” through education for service providers and stronger relationships with the recipients’ families could improve overall quality.
***2. the impact of LTCI on the economic burden of beneficiary families***						
7	Zuchandke et al. (2010) [25]	GENEVA PAPERS ON RISK AND INSURANCE-ISSUES AND PRACTICE	Germany	Quantitative research	This paper analyses the effect of the introduction of compulsory long-term care insurance in 1995 in Germany on the perception of financial security when needing long-term care.	Experience with long-term care had no significant effect before the introduction but a positive effect afterwards. Also, the perception of financial security is found to be increasing with income at both times with similar magnitudes.
8	Schwarzkopf, et al. (2012) [30]	BMC HEALTH SERVICES RESEARCH	Germany	Quantitative research	This study quantified the additional yearly expenditures per dementia patient for various health and long-term care services.	1.male and female dementia patients incurred comparable total costs. However, women accounted for significantly lower health and significantly higher long-term care expenditures. 2.Long-term care spending increased in older age, whereupon health care spending decreased. Thus, at more advanced ages, women incurred greater costs than men of the same age.
9	Yamada et al. (2009) [16]	PSYCHOLOGY HEALTH & MEDICINE	Japan	Quantitative research	This paper evaluated how care manager support (‘social talk,’ ‘information giving’ and ‘reassurance’) affects the burden of family caregivers categorised by caregiver gender and living arrangement.	Care manager support is only effective for female caregivers living with elderly relatives, and is ineffective or works poorly for female caregivers living separately and male caregivers living with elderly relatives.
10	Iwamoto et al. (2010) [21]	JOURNAL OF THE JAPANESE AND INTERNATIONAL ECONOMIES	Japan	Quantitative research	This paper evaluates the role of the public long-term care insurance scheme implemented in Japan in April 2000 on the welfare losses measured in terms of consumption.	1.when households include a disabled family member, household consumption net of long-term care costs do not decrease as much as before the introduction of long-term care insurance. 2.The introduction of social insurance in 2000 helped Japanese households to reduce the welfare losses associated with a disabled family member.
11	Washio et al. (2012) [32]	INTERNATIONAL MEDICAL JOURNAL	Japan	Qualitative research	This paper studies on the care burden among caregivers before and after the introduction of long term care insurance system since 2000 in Japan.	1.Neither the rate of depressive caregivers nor time spent on caregiving decreased under the LTCI. Although the elderly and their caregivers have right to use services under the LTCI, they use only small part of these services which they had the right to use. 2.municipal services and/or informal services should be provided when the elderly and their caregivers cannot afford to use social service under the LTCI.
12	Kim& Lim (2015) [45]	JOURNAL OF PUBLIC ECONOMICS	Korea	Quantitative research	This paper provides empirical evidence on the short-run impact of government subsidies of long-term care.	1.the first-year impact of subsidies for formal home lead to increases in formal long-term care utilization, even when accounting for crowd out of private spending. 2.publicly financed home care may have limited impact among the more able, but that it may be both more cost-effective and beneficial than institutional care for the least able.
13	Choi et al. (2018) [60]	GERIATRICS & GERONTOLOGY INTERNATIONAL	Korea	Quantitative research	This paper examined whether long-term care insurance (LTCI) reduces medical utilization and the burden of medical costs of beneficiaries.	The burden of medical costs for LTCI beneficiaries were significantly reduced compared with non-beneficiaries, despite the rise in medical costs among older adults. The positive effect of LTCI supports continuous implementation and expansion of the LTCI service for non-beneficiaries who require care assistance.
14	Feng et al. (2020) [71]	SOCIAL SCIENCE & MEDICINE	China	Quantitative research	This study examines the effect of long-term care insurance (LTCI) on hospital utilization and expenditures among the elderly in China.	The introduction of LTCI significantly reduces the length of stay, inpatient expenditures, and health insurance expenditures in tertiary hospitals by 41.0, 17.7, and 11.4%, respectively. Cost-effectiveness analysis indicates that every extra 1 yuan spent in LTCI will generate a decrease of 8.6 yuan in health insurance expenditures.
***3. the impact of public LTCI on long-term care providers***						
15	Geyer&Korfhage (2015) [48]	HEALTH ECONOMICS	Germany	Quantitative research	This paper discussed the labor supply decision of family carers and the incentives set by the long-term care insurance.	Estimated a structural model of labor supply and the choice of benefits of family carers. We find that benefits in kind have small positive effects on labor supply. Labor supply elasticities of cash benefits are larger and negative. If both types of benefits increase, negative labor supply effects are offset to a large extent.
16	Geyer, et al. (2017) [53]	FISCAL STUDIES	Germany	Quantitative research	This paper use a structural model of labour supply and the choice of care arrangement to quantify these indirect fiscal effects of informal care.	Informal care by close family members is the main pillar of most long-term care systems. However, due to demographic ageing, the need for long-term care is expected to increase while the informal care potential is expected to decline.
17	Umegaki et al. (2014) [38]	ARCHIVES OF GERONTOLOGY AND GERIATRICS	Japan	Quantitative research	This paper surveyed the care burden of family caregivers, their satisfaction with the services, and whether their care burden was reduced by the introduction of the LTCI care services.	A lower age of caregivers, a more advanced need classification level, and more satisfaction with the services were independently associated with a reduction of the care burden. In Japanese LTCI, the overall satisfaction of the caregivers appears to be relatively high and is associated with the reduction of the care burden.
18	Fu et al. (2017) [54]	JOURNAL OF HEALTH ECONOMICS	Japan	Quantitative research	this paper investigated the spillover effect in two periods: before and after the introduction of the LTCI in 2000 and before and after its major amendment in 2006.	The LTCI introduction has significant and positive spillover effects on family caregivers’ labor force participation and the effects vary by gender and age. In contrast, the LTCI amendment is found to have generally negative spillover effects on their labor force participation.
19	Kondo (2019) [64]	JOURNAL OF THE JAPANESE AND INTERNATIONAL ECONOMIES	Japan	Quantitative research	This paper examines the effect of raising Long-term care insurance (LTCI) payments on employment and wages of workers in the long-term care (LTC) industry.	No increase in the number of employees in the establishments, registered under the LTCI scheme, in municipalities where the regional premium increased. The earnings and working hours of LTC workers did not increase, either.
***4. the influence of LTC system design including nursing style***						
20	Strier,& Werner (2016) [50]	JOURNAL OF AGING & SOCIAL POLICY	Israel	Qualitative research	This article uses a methodology of personal in-depth and focus group triangulation, by which the views of three groups of stakeholders are explored and compared: persons with AD, relatives, and professionals.	The presence of stigmatic self-images among persons with AD or other types of dementia and the absence of such images in relatives’ and professionals’ views of them and of LTCI. However, treatment stigma was found to be primarily associated with eligibility determination procedures. The study concludes that LTCI, even when mandated and almost universal, may also generate welfare stigma due to the ways in which it is implemented.
21	Bascans et al. (2017) [52]	INTERNATIONAL JOURNAL OF HEALTH ECONOMICS AND MANAGEMENT	France	Qualitative research	This paper investigates theoretically how the structure of means-tested public long-term care (LTC) support influences the relationship between LTC insurance and informal care.	The optimal levels of insurance and informal care as well as their relationship are strongly influenced by the waysmeans-tested public support for LTC is structured, which have important implications in terms of public policy for the financing of LTC needs.
22	Tomita et al. (2010) [27]	BMC HEALTH SERVICES RESEARCH	Japan	Quantitative research	This study aimed to clarify the impact of home and community-based services on the hospitalisation and institutionalisation of individuals certified as eligible for long-term care insurance (LTCI) benefits.	1. Users of home and community-based services were less likely than non-users to be hospitalised or institutionalised. 2. For those with relatively light needs, users of day care were also less likely to be hospitalised or institutionalized than non-users. 3.Respite care, rental services for assistive devices and day care are effective in preventing hospitalisation and institutionalisation. 4.home and community-based services contribute to the goal of the LTCI system of encouraging individuals certified as needing long-term care to live independently at home for as long as possible.
23	Seok (2010) [20]	SOCIAL WORK IN PUBLIC HEALTH	Korea	Qualitative research	This paper examines the radical change and impacts on service financing, provision, and governance from the introduction of the long-term care insurance for the elderly in Korea.	1.The long-term care service has transformed from the very selective service applicable only to low-income groups to a universal service for all income groups. 2.The service provision method has been changed from the provision by nonprofit organizations entrusted by the state under a monopolistic commission arrangement in the past to a new open-service provision arrangement in which free competition among service providers in service market and consumers’ choice will be emphasized.
24	Kim et al. (2013) [37]	HEALTH POLICY	Korea	Quantitative research	This study examined the patterns of and factors associated with public long-term care (LTC) utilization among older LTCI beneficiaries in Korea, with special attention to the policy for subsidizing the co-payments of lower income populations.	About 5.48% of older adults in 2010 utilized the LTC provided under the Korean public LTCI among which about 26.1% received a subsidy. The findings imply the subsidy policy promotes equity of access to public LTC services. Further evaluation is necessary on the impact of the policy on the effectiveness of LTC utilization by socially marginalized populations.
25	Hyun et al. (2014) [43]	BMC HEALTH SERVICES RESEARCH.	Korea	Quantitative research	This study examines the effects of long-term care insurance (LTCI) on the length of stay (LoS) of senior citizens under the national health insurance of Korea.	the LoS of LTCI users is 1.27 days greater than that of non-LTCI users, but the LoS of level 1 and level 2 beneficiaries decreases by 8.35 and 2.84 days, respectively, whereas the LTCI does not reduce the LoS of level 3 beneficiaries. Thus, recommend a modification in the LTCI system that facilitates the use of long-term care institutional services by level 3 beneficiaries to promote home-based care services instead of the institutional care services.
26	Kim et al. (2019) [62]	JOURNAL OF AGING & SOCIAL POLICY	Korea	Quantitative research	This study analyzed the association between living arrangement and caregiver type with institutionalization in different LTCI grade beneficiaries using from 2008 to 2013.	Higher likelihoods of institutionalization were found in individuals living with a non-family member compared to individuals living with their spouses. Individuals without a caregiver or with a paid caregiver were also more likely to experience institutionalization than individuals with a spouse primary caregiver.
27	Zhang&Yu (2019) [65]	INTERNATIONAL JOURNAL OF ENVIRONMENTAL RESEARCH AND PUBLIC HEALTH	China	Quantitative research	This paper explored the outcomes and evaluate the performance of the LTCI policy in the Chinese pilot cities	The relationship between living location and number of children of the family and the outcomes and performance of the LTCI policy in the pilot cities was significant. The rest of the factors showed no significance with the implementation of the LTCI in Chinese pilot cities.

Firstly, the impact of public LTCI on the health of beneficiaries. As the first country with formal legislation and public LTCI, Germany has made remarkable achievements in LTCI reform. The physical health of the beneficiaries of the public LTCI has improved significantly for the most part, especially those receiving good home care (Buscher, et al. 2010). The changes in Japan (Olivares-Tirado et al. 2012; Ohwaki, et al. 2009; Chen et al. 2013) and South Korea (Lee et al. 2014 ; Sohn et al. 2020), the two Asian countries that decided on public LTCI in 2000 and 2008 respectively, have also been remarkable.

Secondly, the impact of LTCI on the economic burden of beneficiary families, which is an inevitable problem that must be solved in the process of sustainable development of public LTCI. For instance, after the implementation of public LTCI, German people’s perception of financial security also increases with the increase of income (Zuchandke et al. 2010), but there is a gender and age difference. When the age is older, women spend more than men of the same age (Schwarzkopf, et al. 2012). The introduction of the public LTCI in 2000 helped Japanese families reduce welfare losses associated with members of families with disabilities (Yamada et al. 2009; Iwamoto et al. 2010; Washio et al. 2012). In Korea, the burden of medical costs for LTCI beneficiaries was significantly reduced compared to non-beneficiaries, although medical costs for the elderly increased (Kim& Lim, 2015; Choi et al. 2018). In China, the introduction of public LTCI has greatly reduced the length of stay, hospitalization costs and medical insurance costs in tertiary hospitals by 41.0, 17.7 and 11.4%, respectively. The cost-benefit analysis shows that each additional dollar spent at LTCI reduces health insurance spending by $8.6(Feng et al. 2020).

Thirdly, the impact of public LTCI on long-term care providers, i.e., the labor market. German scholars Geyer designed a structural model of labor supply and family caregiver welfare choices and found that benefits in kind had a small positive effect on labor supply (Geyer&Korfhage, 2015; Geyer, et al. 2017). Japanese researchers found that the introduction of public LTCI had a significant positive spillover effect on the labor force participation of family caregivers, and the effects varied by gender and age (Umegaki et al. 2014; Fu et al. 2017; Kondo, 2019).

Finally, the influence of LTC system design, including nursing style, is discussed. The influence of public LTCI on the choice of long-term care methods in various countries, such as home care, community care, and institutional care, is restricted by income level, family environment, and other factors (Strier,& Werner , 2016; Bascans et al. 2017; Tomita et al. 2010; Seok, 2010; Kim et al. 2013; Hyun et al. 2014; Kim et al. 2019; Zhang&Yu,2019).

### Challenge of public LTCI

As a new type of insurance coexisting with endowment insurance and medical insurance, LTCI system has experienced different degrees of difficulties and challenges in the development process of different countries, which will affect the long-term sustainability and stability of LTCI system development (as shown in Table 5).

**Table 5:** The Challenge of public LTCI

Rank	Author	Journal	Country	Method	Objective / feature	Key findings
***1. challenges from the sustainability of LTCI financing***						
1	Schut&van den Berg (2010) [23]	SOCIAL POLICY & ADMINISTRATION	Netherlands	Qualitative research	This paper examined the past experiences, current deficiencies and future prospects of LTC financing in the Netherlands.	The success of the LTCI reforms heavily depends on the definition of entitlements, the accuracy of needs assessment and the feasibility of determining appropriate client-based budgets..
2	Rothgang (2010) [24]	SOCIAL POLICY & ADMINISTRATION	Germany	Qualitative research	This paper discusses what should be the focus of the second reform of German public LTCI system.	the focus of the second reform of German public LTCI system should concentrated on quality improvements, care management and careful adjustments of benefits. Financing issues are of particular concern such as increasing in the contribution rate.
3	Nadash et al. (2018) [57]	GERONTOLOGIST	Germany	Qualitative research	The study reviews legislative and programmatic changes of LTCI, using program data, as well as legislative documents and program reports.	1.The program is widely accepted among citizens and has achieved many of its original goals: ensuring access to long term services and supports and reducing reliance on the locally-funded safety-net social assistance program, which can be used to cover nursing home costs. 2.Recent reforms has addressed financing issues by increasing premiums, introducing subsidies for the purchase of private insurance, and creating a “demographic reserve fund.” 3.the program provides evidence for the financial viability of a social insurance model, although longer-term challenges may yet arise..
4	Tamiya et al. C (2011) [28]	LANCET	Japan	Qualitative research	Lessons from Japan’s long-term care insurance policy	There has been an increase in families’ access to formal care at lower cost, but results for the well-being of caregivers have been mixed. The system’s successful challenges include dissatisfaction with home care, the provision of necessary support to home caregivers, and financial sustainability.
5	Kato(2018) [59]	JAPAN AND THE WORLD ECONOMY	Japan	Quantitative research	This paper explores the impact of population aging on the Japanese public long-term care insurance (LTCI) within a numerical dynamic general equilibrium model with multiple overlapping generations.	In order to reduce future burdens in the LTCI, an increase in co-payments is most preferable, rather than an earlier starting age of contribution in the longer duration with lower annual burdens, or a shift of the cost to the public sector with a very high consumption tax.
6	Wang et al. (2018) [55]	INTERNATIONAL JOURNAL OF ENVIRONMENTAL RESEARCH AND PUBLIC HEALTH	China	Quantitative research	This study collected data from a household survey conducted in Qinghai and Zhejiang on a sample of 1842 households, and explored the determinants of demand for LTCI.	Price, age, education status, and income were significantly associated with demand for LTCI. Most pilot cities were found to mainly rely on Urban Employees Basic Medical Insurance funds as the financing source for LTCI. financing is one of the greatest challenges in the development of China’s LTCI.
7	Zhang et al. (2020) [68]	SUSTAINABILITY	China	Quantitative research	Based on the International Labor Organization (ILO) financing model from the perspective of fund balance, an overall simulation model and a Monte Carlo simulation are used to estimate the contribution rate of LTCI for disabled elderly from 2020 to 2050 in China.	1.The total financial demands will increase sharply from 538.0 billion yuan in 2020 to 8530.8 billion yuan in 2050. Of that total, 80.2% will be required in urban areas. 2. the per capita financial demands of care in urban and rural areas in 2050 will be approximately six times and 11 times higher than in 2020, respectively. 3.the overall contribution rate of LTCI in China will increase sharply from 1.46% in 2020 to 5.14% in 2050, an increase of about 3.5 times. By comparison, the contribution rate in 2020 will be close to 1.33% in Japan in 2015 and 1.40% in Germany in 2010.
***2. challenges from design flaw of LTCI system***						
8	Ariizumi, (2008) [15]	JOURNAL OF HEALTH ECONOMICS	USA	Quantitative research	This paper investigate the effects of two common eligibility criteria of LTC programs: means-tested and health-based programs.	1.Publicly provided health-based LTC crowds out the medical spending among low health individuals. 2.Means-tested programs lead to higher initial spending on medical care and consumption goods among middle-wealth individuals.
9	Zhu& Osterle (2019) [63]	INTERNATIONAL JOURNAL OF HEALTH PLANNING AND MANAGEMENT	China	Qualitative research	This paper estimates the prevalence of LTC needs and analyzes the impact of the LTCI pilots on access.	Future policy experimentation on LTCI reform in China needs to address the following pressing policy issues: expanding the coverage of LTCI; narrowing rural-urban disparities in access; improving access for vulnerable subpopulations; and reducing the heavy reliance on institutional care.
10	Kang et al. (2012) [29]	JOURNAL OF KOREAN MEDICAL SCIENCE	Korea	Qualitative research	This paper discussed LTCI’s eligibility qualifications and the certification process based on functional disability, benefits and coverage of community-based and institutional care in Korea.	The lack of coordination between the health and long-term care sectors, limited consideration of physicians’ assessments in the certification process, inadequate provision of health services in long-term care facilities, and overlapping and inefficient use of care resources act as barriers to providing comprehensive healthcare for older beneficiaries.
11	Chon (2013) [35]	JOURNAL OF SOCIAL SERVICE RESEARCH	Korea	Qualitative research	This study aims to understand how National Health Insurance Corporation (NHIC) staff and home-visiting service providers experienced and evaluated the new service delivery system.	Korea’s new LTCI service delivery system faces challenges and that a more active role for the Korean government, especially regarding the introduction of a proper care management system, is needed to address the issues.
12	Chon (2014) [42]	SOCIAL POLICY & ADMINISTRATION	Korea	Qualitative research	This study examined how the Korean welfare state has coped with the increasing LTC needs of older people caused by introducing and implementing a new LTCI system and reforming it.	LTCI was designed to meet limited objectives, such as providing minimal coverage and affording private for-profit market forces a predominant role in the provision of LTCI services.
***3. challenges from traditional social concepts or family relationships***						
13	Costa-Font (2010) [26]	OXFORD REVIEW OF ECONOMIC POLICY	Europe	Quantitative research	The empirical analysis of the paper exploits cross-country and sub-group variability of a representative database of European Union member states, containing records on LTC coverage and family structure.	A negative association between family ties and expected coverage of LTC for different sub-samples. Policy implications suggest that widespread expansion of LTC coverage might need to accommodate existing familistic cultural norms to avoid insurance crowding out.
14	Chon (2012) [33]	ASIA PACIFIC JOURNAL OF SOCIAL WORK AND DEVELOPMENT	Korea	Qualitative research	This paper presents an overview of the key issues that were involved in designing and implementing the new Korean system and the lessons that have been learned.	Although the government reformed the long-term care system, a number of new challenges have emerged, such as its limited coverage and the unethical behavior of service providers.
15	Kim&Choi (2013) [36]	AGEING & SOCIETY	Korea	Qualitative research	This paper aims to analyse the nature of LTCI in South Korea and to examine whether its introduction(2008) could mean a divergence from thepath of both developmentalism and Confucianism.	The regulatory role of the government and concerns about the costs of LTCI are regarded as a developmentalist legacy, whereas Confucian legacies seem to be withering away since LTCI shifts care responsibility from the family to the state. However, the study found that the state has difficulty in regulating the market and costs, and deeply embedded familialism seems difficult to overcome.
16	Ha et al. (2017) [51]	JOURNAL OF SOCIAL SERVICE RESEARCH	Korea	Quantitative research	This study examined whether the awareness of public long-term care insurance (LTCI) was related to South Koreans’ financial, psychosocial, and physical preparations for later life.	1. being aware of public LTCI increased preparation for later life in financial, psychosocial, and physical domains. 2.participants in the negative status group had higher scores for psychosocial and physical preparation than individuals who perceived themselves as being in relatively better situations. 3.the policy makers and practitioners should include education on LTCI and the risks it targets across various preparation domains as well as account for differences in perception and preparation among sub-populations of middle-aged adults in order to ensure effective social policies for the elderly population.
17	Ayalon (2018) [58]	HEALTH & SOCIAL CARE IN THE COMMUNITY	Israel	Qualitative research	The present study aimed to evaluate the perspectives of older adults, their family members and home care workers regarding the LTCI.	How even though the NII workers are engaged with various stakeholders, they often lack direct contact with older adults, their family members and paid home care workers: those most directly influenced by the LTCI.
***4. challenge of balancing fairness and efficiency***						
18	Bakx&Schut& van Doorslaer (2015) [47]	INTERNATIONAL JOURNAL OF HEALTH ECONOMICS AND MANAGEMENT	Dutch	Qualitative research	This paper examined the feasibility of appropriate risk adjustment in LTC insurance.	Prior health care expenditures are also important in reducing predicted losses for subgroups of health care users. Nevertheless, incentives for risk selection against some easily identifiable subgroups persist. Moreover, using prior utilization and expenditure as risk adjusters reduces incentives for efficiency, creating a trade-off between equity and efficiency.
19	Park (2015) [49]	INTERNATIONAL JOURNAL FOR EQUITY IN HEALTH	Korea	Quantitative research	The Aday-Andersen model was used as a conceptual model to examine the extent to which equity in the use of long-term care has been achieved in Korea.	1.those who rated his or her health to be fair, good, and very good, had no limited activities, were disabled, and had insurance coverage were more likely to use long-term care services, respectively. 2.the introduction of a national long term care insurance program did not yield a fully equitable distribution of services. 3. Long-term care reforms in Korea should continue to concentrate on expanding insurance coverage and reducing the inequities reflected in disparities in consumer cost sharing and associated patterns of utilization across plans.
20	Saito et al. (2018) [56]	PLOS ONE	Japan	Quantitative research	This study examined income-based inequalities in caregiving time and depressive symptoms in Japanese older family caregivers under a public, universal long-term care insurance (LTCI) system.	1.A Poisson regression model revealed that caregivers in lower income groups (compared to those in the highest) were 1.32 to 1.95 and 1.63 to 2.68 times more likely to engage in > = 36 and > = 72 h/week of caregiving, respectively. 2.an interaction effect of income by caregiving role indicated no significant difference in inequality between caregivers and non-caregivers (*p* = .603).
21	Yang et al. (2020) [67]	RESEARCH ON AGING	China	Quantitative research	This paper discussed the effects of piloted LTCI on equity and efficiency in financing in Qingdao city.	There remain sizable disparities in financial burden among insurance participants, despite an emphasis on ensuring equitable access to care. Although the insurance brought cost savings to the health care sector, the LTC providers are incentivized to provide care at the least cost, even when such care is deemed inadequate due to the fixed payment for their services.

Firstly, the challenges from the sustainability of LTCI financing, which is a core issue for LTCI’s long-term development. For instance, the success of the Netherlands LTCI reform depends to a large extent on the accuracy of the LTCI needs assessment and the proper budgeting of LTC costs (Schut& van den Berg,2010). Germany’s public LTCI has undergone several reforms, as has Japan (Tamiya et al. 2011; Kato,2018), focusing on solving the financing problem through higher contribution rates and the creation of a “population reserve Fund” since 1995(Rothgang,2010; Nadash et al. 2018). Financing is one of the greatest challenges in the development of China’s LTCI system and most pilot cities mainly rely on Urban Employees Basic Medical Insurance funds as the financing source for LTCI (Wang et al.2018; Zhang et al.2020).

Secondly, the challenges from design flaw of LTCI system, including certification. For instance, publicly provided health-based LTC crowds out the medical spending among low health individuals in USA (Ariizumi, 2008),while future policy experimentation on LTCI reform in China needs to address the some policy issues such as expanding the coverage of LTCI and narrowing rural-urban disparities in access (Zhu& Osterle,2019). The lack of coordination between the health sector and the long-term care sector is a weak point in the development of Korea’s public LTCI (Kang et al.2012; Chon, 2013,2014).

Thirdly, challenges from traditional social concepts or family relationships. For example, Whether in Europe (Costa-Font, 2010) or Israel (Ayalon, 2018), family relationships or family culture are correlated with the benefit coverage of LTCI. This is especially true in South Korea, which is heavily influenced by traditional East Asian culture. Many Korean studies found that the state has difficulty in regulating the market and costs, and deeply embedded familialism seems difficult to overcome (Chon, 2012; Kim& Choi2013; Ha et al.2017).

Finally, how to balance fairness and efficiency is also a big challenge for the future development of public LTCI. For example, Dutch using prior utilization and expenditure as risk adjusters reduces incentives for efficiency, creating a trade-off between equity and efficiency (Bakx et al.2015).Long-term care reforms in Korea should continue to concentrate on expanding insurance coverage and reducing the inequities reflected in disparities in consumer cost sharing and associated patterns of utilization across plans (Park, 2015). There remain sizable disparities in financial burden among insurance participants, despite an emphasis on ensuring equitable access to care in China (Yang et al.2020) and Japan (Saito et al.2018).

### Relationship between public LTCI and private LTCI

As shown in Table 6, Brown&Finkelstein (2008) ‘s study published in AMERICAN ECONOMIC REVIEW in 2008 was the first classic paper in the world to discuss the relationship between PUBLIC LTCI and private LTCI. Two years later, the difference between public nursing institutions and private nursing institutions in the Long term care effect of disabled elderly after the formal launch of public LTCI system in Japan since 2000 was compared (Yoshioka et al.,2010). Their study found that private LTCI plays an important role in promoting the use of care services, but the quality of care may be problematic. In the following years, some scholars conducted in-depth discussions on the crowding out effect of public LTCI and private LTCI (Cremer&Pestieau,2014; Costa-Font&Courbage,2015), cooperation mode (Schmitz& Giese,2019) and other aspects.

**Table 6:** The Relationship between public LTCI and private LTCI

Rank	Author	Journal	Country	Method	Objective / feature	Key findings
1	Brown&Finkelstein (2008) [14]	AMERICAN ECONOMIC REVIEW	USA	Qualitative research	This paper discussed the interaction of public and private LTCI	Although the private LTCI market in the United States is still developed, it can be a useful supplement to the public LTCI in the future
2	Yoshioka et al. (2010) [18]	GERIATRICS & GERONTOLOGY INTERNATIONAL	Japan	Quantitative research	This study compared differences in the effect of the public care management agencies and private care management agencies on LTCI service use.	1.Public care management agencies favored younger subjects, male subjects and people with a higher need for care than private agencies. 2.The utilization of community-based long-term care service was significantly greater among beneficiaries managed by private agencies than those managed by public agencies. 3.Private care management agencies play an important role in promoting the use of care services, but their quality of care plans might be questionable.
3	Shen et al. (2014) [39]	SOCIAL WORK IN HEALTH CARE	China	Quantitative research	This paper explored long-term care insurance (LTCI) plans in China and the factors associated with each plan’s contribution rate.	Public LTCI may be more popular whether in terms of participation or contribution. Policymakers should develop public LTCI as a solid foundation and improve private LTCI as a substitute to meet the urgent LTC needs in China.
4	Cremer&Pestieau (2014) [41]	INTERNATIONAL TAX AND PUBLIC FINANCE	Belgium	Quantitative research	This paper study the role of social long-term care (LTC) insurance when income taxation and private insurance markets are imperfect.	First, whereas in the linear case a subsidy of private LTC insurance is desirable, it is not in the nonlinear case (at least at the margin). Second, the desirability of public provision of LTC services depends on the way the income tax is restricted.
5	Costa-Font&Courbage (2015) [44]	HEALTH ECONOMICS	European	Quantitative research	This paper aims to study the motivational crowding-out hypothesis “public sector funding’ and family support’ crowd out individual incentives to seek insurance” by developing a theoretical model and representative European survey data.	The theoretical model predicts that, when informal care is treated as exogenously determined, expectations of both state support and informal care can potentially crowd out LTC insurance expectations. But no robust evidence of public sector crowding out.
6	Schmitz&Giese (2019) [61]	GENERATIONS-JOURNAL OF THE AMERICAN SOCIETY ON AGING	USA	Qualitative research	This article lays out ways in which the public and private markets could work together to offer insurance coverage.	The combination of private and public LTCI can provide a way to plan and finance risks that require paid-for long-term care (LTC) services.

In addition, Before China officially launched the pilot system of public LTCI in 2016, some Chinese scholars discussed what roles public LTCI and private LTCI should play in response to China’s huge long-term care needs. For instance, Shen& Li (2014) conducted a cross-sectional survey of 814 residents (ages 18–59) and found that public LTCI was likely to be more popular in terms of participation and contribution. Factors associated with public LTCI demand are medical costs, household income, and for private LTCI, these factors include the proportion of living expenses, concerns about future care, and medical costs. Therefore, policy makers should develop public LTCI as a solid foundation and perfect private LTCI as an alternative.

## Discussion

In order to deal with the aging population and its long-term nursing costs, the research on LTCI has become the international academic frontier and hot topic. Judging from the current development of global LTCI system, the development of private LTCI is still in its infancy. Countries including Germany, the United States, Japan, South Korea and China all focus on the construction and sustainable development of public LTCI system. In practice, although the LTCI system originated in the Netherlands, Germany (1995) and Japan (2000) are the first countries in the world and Asia to legislate public LTCI, and have been carrying out many reforms and improvements for more than 20 years. Since 2016, China, as the country with the largest aging population in the world, began to pilot public LTCI, which has a great impact on people’s daily life and academic circles. This paper attempts to systematically sort out the relevant research literature on public LTCI, and to explore the continuous reform and improvement of LTCI system in Germany, Japan and South Korea, so as to seek experience and promote the further sustainable development of China’s public LTCI. This is also a further thinking to cope with the impact of covid-19 and other similar public health events on subsequent LTC services.

Firstly, a preliminary review of relevant literature was done. Of the 59 papers selected, 38 were quantitative and 21 were qualitative. Among them, quantitative research is mainly carried out from two perspectives. One is empirical research through microscopic investigation data, such as logistic regression analysis. The second is to discuss the long-term sustainable development of public LTCI caused by financing problems by comparing the financing of public LTCI with the macro data of cost demand. With the passage of time, there are more and more researches on public LTCI, which shows that the development of public LTCI is paid more and more attention by governments and academic circles. The research on public LTCI is mainly concentrated in Korea, Japan and Germany. Relevant research has begun to emerge in China since 2016. It also shows that the research on public LTCI can be divided into three climaxes in terms of time, namely, 2009–2010, 2014–2015 and 2017–2019. The first climax period (2009–2010) is the next 2 years after the official implementation of public LTCI (2008) in South Korea, so there are a lot of studies on Korean public LTCI during this period. The second climax period (2014–2015) is the second 2 years of the fifth reform of public LTCI in Japan, during which there are many relevant studies in Japan. The third climax period (2017–2019) is driven by China’s public LTCI pilot project in 15 cities in 2016.

Secondly, from the comparison of public LTCI systems in different countries, By comparing the development of public LTCI in the Netherlands, Germany, South Korea and Japan, we find that the structural design of public LTCI, including fund-raising and qualification procedures, has different characteristics in different countries (Campbell et al. 2010; Bakx et al. 2015;Courbage et al. 2020;Rhee et al. 2015;Chandoevwit&Wasi,2020). In order to operate stably and sustainably, public LTCI must adapt to local conditions and reform continuously.

Thirdly, the social and economic impact of public LTCI on population aging is enormous. Firstly, The physical health of the beneficiaries of the public LTCI has improved significantly, especially those receiving good home care in Germany,Japan,and Korea (Buscher, et al. 2010; Olivares-Tirado et al. 2012; Ohwaki, et al. 2009; Chen et al. 2013) and South Korea (Lee et al. 2014; Sohn et al. 2020). Secondly, the introduction of the public LTCI in 2000 helped families reduce economic welfare losses associated with members of families with disabilities (Yamada et al. 2009; Iwamoto et al. 2010;Washio et al. 2012;Kim& Lim, 2015; Choi et al. 2018). for example, the introduction of public LTCI in China has greatly reduced the length of stay, hospitalization costs and medical insurance costs in tertiary hospitals by 41.0, 17.7 and 11.4%, respectively (Feng et al. 2020).

Finally, LTCI system has experienced different degrees of difficulties and challenges in the development process of different countries, which will affect the long-term sustainability and stability of LTCI system development. Among them, the challenges from the sustainability of LTCI financing, which is a core issue for LTCI’s long-term development (Schut& van den Berg,2010; Tamiya et al. 2011; Kato,2018; Rothgang,2010; Nadash et al. 2018). Financing is one of the greatest challenges in the development of China’s LTCI system (Wang et al.2018; Zhang et al.2020).

The literature review of this paper gives us some policy implications. First of all, learning from the practical experience of LTCI system in the world, such as structural design and financing mode, will contribute to the in-depth development of China’s public LTCI system. However, the literature review also makes us realize that, restricted by different factors in different countries, the long-term care system is a long-term process, which needs continuous reform (Bakx et al. 2015;Chandoevwit&Wasi,2020) in order to truly meet the growing and changing long-term care needs, especially in China, which has the largest elderly population in the world. Secondly, it is always the core of the stable and sustainable development of public LTCI to solve the financing problem of public LTCI. However, the breakthrough of traditional social concept and family concept, the balance of fairness and efficiency, are very important factors that affect the sustainable development of public LTCI, especially Japan, South Korea, and China, which are deeply influenced by traditional culture (Costa-Font, 2010; Chon, 2012; Kim& Choi,2013; Ayalon, 2018). Perhaps, while steadily promoting public LTCI, it is wise to actively develop private LTCI market (Schmitz& Giese,2019).

In general, Germany, Japan, and other countries have their characteristics in the specific implementation policies of public LTCI, such as fundraising. For example, German LTCI law emphasizes two essential principles, namely, “in the home care priority” and “prevention and rehabilitation priority.” All medical insurance policyholders, as long as is an adult, regardless of men and women’s health and age, must attend the LTCI. On fundraising, the difference according to individual income level presses different proportions to execute personal pay costs. Spending on Long-term care services accounted for about 1% of GDP in 2018. Overall, there are two types of public LTCI (Long-Term Care Insurance) systems in different countries at present. The first is the Nordic “welfare state” model of the comprehensive general welfare, which is not suitable for China’s specific social conditions. The second is the “corporatist-welfare” mode of universal coverage, which emphasizes the combination of comprehensive coverage and mutual benefit, which is meet China’s current “peer-to-peer” rights and obligations of social insurance concept consistent, but there are some risks such as the cost burden. Therefore, China’s future public LTCI development should choose a unique path suitable for its social conditions.

## Data Availability

The original data that If the reader has a personal request, I will provide it to him.

## Abbreviations

LTC: Long-Term Care
LTCI: Long-Term Care Insurance
Public LTCI: Public Long-Term Care Insurance
Private: LTCI Private Long-Term Care Insurance
ADL: Activities of daily living
CASP: Critical Appraisal Skills Programme
